# Physicochemical Composition and Nutritional Properties of Deer Burger Enhanced with Healthier Oils

**DOI:** 10.3390/foods9050571

**Published:** 2020-05-04

**Authors:** Marcio Vargas-Ramella, Paulo E. S. Munekata, Mirian Pateiro, Daniel Franco, Paulo C. B. Campagnol, Igor Tomasevic, Rubén Domínguez, José M. Lorenzo

**Affiliations:** 1 Centro de Educação Superior da Região Sul—CERES da Universidade do Estado de Santa Catarina, Chapecó, Santa Catarina 89.800-000, Brazil; marcio.ramella@hotmail.com; 2 Centro Tecnológico de la Carne de Galicia, Rúa Galicia Nº 4, Parque Tecnológico de Galicia, San Cibrao das Viñas, 32900 Ourense, Spain; paulosichetti@ceteca.net (P.E.S.M.); mirianpateiro@ceteca.net (M.P.); danielfranco@ceteca.net (D.F.); rubendominguez@ceteca.net (R.D.); 3 Department of Food Science and Technology (DTCA), Universidade Federal de Santa Maria, Santa Maria CEP 97105-900, Rio Grande do Sul, Brazil; paulocampagnol@gmail.com; 4 Department of Animal Source Food Technology, Faculty of Agriculture, University of Belgrade, Nemanjina 6, Belgrade 11080, Serbia; tbigor@agrif.bg.ac.rs

**Keywords:** lipid oxidation, healthy meat product, tiger nut, chia, linseed, sensorial analysis

## Abstract

Deer meat is characterized by low fat and cholesterol contents and high amounts of protein and polyunsaturated fatty acids. In this regard, the aim of this work was to assess the influence of pork backfat substitution by healthier oils on chemical composition, fatty acid profile, texture profile and sensory analysis of deer burger. In addition, pH, color parameters and lipid oxidation were evaluated at 0, 6, 12 and 18 days of storage. For this study, four different treatments of deer burgers—100% pork backfat, 100% tiger nut oil, 100% chia oil, and 100% linseed oil—were elaborated. The fat replacement reduced fat and protein contents and increased moisture amounts, whereas ashes and texture parameters of deer burgers were not affected. Fatty acid profile was significantly improved with the animal fat replacement. In this regard, a significant decrease in saturated fatty acids was found in all reformulated batches, whereas in chia and linseed burger samples a dramatic increase in polyunsaturated fatty acids, omega-3 content and a reduction of n-3/n-6 ratio was observed. In the deer burger prepared with tiger nut oil a significant increase in monounsaturated fatty acids was found. Another important aspect is that the replacement of animal fat by tiger nut or linseed oil emulsion did not affect the global acceptance of deer burgers. Regarding color parameters, redness was the most affected during the whole display presenting a reduction around 50% after 18 days of storage. On the other hand, thiobarbituric acid reactive substances (TBARS) values were also affected by fat replacement and storage time, observing the highest values (2.43 mg MDA/kg) in deer burgers prepared with chia at the end of refrigerated period. Finally, from a commercial point of view, the possibility of making claims such as “low fat burgers”, “reduced saturated fat” or “high content of omega-3” makes the reformulated burgers more attractive to the consumer.

## 1. Introduction

Based on estimations by the World Health Organization (WHO), approximately 462 million adults worldwide were underweight, while 1.9 billion were either overweight or obese [1]. In the last few years, most countries have seen an unhealthy diet high in saturated fatty acids (SFA) contributing to the overweight population and chronic disorders, particularly ischemic heart disease and stroke [1,2,3,4]. To reduce all forms of malnutrition and the incidence of such diseases, recommendations for optimal intake of total fat and unsaturated fatty acids have been proposed by many governments [2,3,5].

Food reformulation is included in nutrition action plans of many countries, and currently, almost every European Union (EU) country has at least one plan or program on food/nutrition and health for salt-fat-sugar reduction [2]. Additionally, the increased intention of consumers to buy healthier meat products with favorable nutritional information has given rise to the reformulation of some traditional meat products [6,7]. From the consumer point of view, innovation should not assume the loss of sensory characteristics of the traditional product [8]. Thus, meat product reformulation using vegetable or marine oils as animal fat replacers [9,10,11,12,13,14] could be a good strategy. Among the multiple vegetable oils, chia and linseed oils are characterized by having a high content of α-linolenic acid (around 60%), high PUFA/SFA ratio (up to 7.40) and a n-6/n-3 ratio that can reach 0.30, which are used in several studies [8,9,15,16]. In contrast, tiger nut is an untapped source of health food with highly nutritional values and a relevant source of monounsaturated fatty acids (MUFA) such as oleic acid (67%–69%) [9,17,18,19].

Spain is the second largest world producer of deer venison which mainly exports meat from hunted animals. The official data on deer harvested seems to underestimate the real production that accounted for 235,000 cervids harvested in 2018 (144,134 deer, 66,737 roe deer and 24,337 fallow deer; MAPA [20]). At a conservative estimate of 50 kg/carcass, 11,750 tons of deer meat are exported (co-products not included) according to industry estimates, but even the official figures reach 14,400 tons, which is composed of 11,530 tons of red deer venison, 1668 tons of roe deer, and 1241 tons of fallow deer venison [20]. In addition, deer (venison) is raised under natural conditions and has enjoyed a rise in popularity among consumers in recent years [21,22]. It is also worth noting that deer meat has excellent nutritional characteristics. This meat has low fat and cholesterol contents and high amounts of protein, essential amino acids and minerals [21,23,24,25,26]. Additionally, the fatty acids profile of the deer meat is characterized by high amounts of PUFA (43.80 g/100 g total fatty acids) and long-chain n-3 [9,24,25,26], which have positive effects on human health.

Among the meat products, burgers are consumed worldwide, usually produced from 70% to 75% of meat and 25% to 30% of fat [27,28]. Other studies have shown that beef burger contains from 9% to 20% animal fat in formulation, with high SFA content (31%–42%) [16,29,30,31]. Pork backfat is commonly used as a fat source in meat products. However, its nutritional quality is questioned, since it contains an unfavorable fatty acid profile with a high level of SFA and high n-6/n3 ratio [16,32,33,34]. Thus, several researchers indicated that the partial or total replacement of pork back fat by vegetable or marine oils resulted in a reduction of both, total fat and SFA contents [9,10,12,14,31]. Additionally, the content of cholesterol showed a significant reduction with the animal fat replacement by vegetable oils [14].

Thus, due to the nutritional quality that vegetable oils can provide to consumers by increasing the α-linolenic or oleic acid in meat products with the animal fat replacement, the present study aimed to reformulate deer burgers to make them healthier through the total replacement of pork back fat by linseed, chia or tiger nut oils emulsions and evaluate its physicochemical, nutritional and sensory characteristics.

## 2. Materials and Methods

### 2.1. Preparation of Vegetable Oil Emulsion

The study was performed in the Centro Tecnolóxico da Carne (CTC) de Galicia (San Cibrao das Viñas, Spain). In order to prepare the vegetable oil emulsion with Prosella (Prosella VG NF4, Colin Ingrédients, Mittelhausen, France), its elaboration was performed one day before the processing of burgers [31,35]. The Prosella powder was composed of jellifying agents (calcium sulphate and sodium alginate), wheat glucose syrup (7.4%), a stabilizer (disodium diphosphate, added P_2_O_5_: 9.58%) and an antioxidant (sodium ascorbate), which retain oils in its structure and can be used as animal fat replacer. For oil emulsions preparation, water (56 g/100 g) and vegetable oil (37.3 g/100 g) were mixed for 1 min in a bowl cutter (Sirman, mod C15VV, Marsango, Italy). The Prosella powder (6.7 g/100 g) was added and homogenized during 3 min and then left to rest for 2 h, then the mixture was refrigerated at 4 °C until needed.

### 2.2. Deer Burgers Manufacture

Four different formulations were processed: control (containing pork back fat as lipid source (3 g/100 g; formulation with low amount of fat added)) and three experimental batches in which animal fat was replaced by vegetable oil emulsion (immobilized in prosella gel) (3 g/100 g) (tiger nut oil (TIG); chia oil (CHI); linseed oil (LIN)). The other ingredients used in all formulations were deer lean meat (87.8 g/100 g), salt (1.2 g/100 g) and water (8 g/100 g). Prime cuts of foreshank from hunted deer supplied by Cárnicas Dibe (Cáceres, Spain) and pork fat were used in burger processing. All visible fat and connective tissue were removed manually from the meat. For burger processing, firstly primal cuts of foreshank and pork fat and/or vegetable oil emulsions were ground through an 8 mm and 6 mm diameter mincing plate in a refrigerated mincer machine (La Minerva, Bologna, Italy), respectively. The meat batter was mixed together with the remaining ingredients until complete homogenization and shaped in a burger format. The different treatments were shaped (10 cm diameter and 1 cm height) in a manual burger machine. A total of 2 kg of mass was prepared, resulting in 25 burgers in each treatment and weighing 80 g each.

After processing, burgers were packed under modified atmosphere (80% O_2_ and 20% CO_2_) in 300 mm thick PET-EVOH-PE trays, sealed with multilayer PE-EVOH-PE film (74 mm thick, permeability < 2 mL/m^2^ bar/day (Viduca, Alicante, Spain)) using a heat sealer (LARI3/Pn T-VG-R-SKIN, Ca.Ve.Co., Palazzolo, Italy). The samples were stored at 2 ± 1 °C under light, simulating the conditions from the supermarket and analyzed at 0, 6, 12 and 18 days of storage (proximate composition, fatty acids, texture profile analysis and sensory analysis on day 0; pH, color parameters and oxidative stability on days 0, 6, 12 and 18). On sampling days, twenty samples (five for each batch) were collected and evaluated for their proximate composition, physicochemical parameters, fatty acids profile and sensory characteristics. The entire experiment was replicated in two different weeks.

### 2.3. Proximate and Physicochemical Analysis of Deer Burgers

#### 2.3.1. Proximate Composition, Carbohydrates and Energy Content

The proximate composition of the different deer burger treatments were evaluated according to International Organization for Standards (ISO), for protein [36], moisture [37] and ash [38] content, while total fat was determined according to the Approved Procedure Am 5–04, established by the American Oil Chemists’ Society [39].

#### 2.3.2. Color and pH

Color parameters (L*—brightness, a*—greenness/redness and b*—blueness/yellowness) were measured in the CIELAB space using a portable colorimeter (CR-600d, Minolta Co. Ltd., Osaka, Japan). The device was set to pulsed xenon arc lamp, 10° viewing angle geometry, and 8 mm aperture. The pH was measured in the deer burgers using a digital pH-meter (Hanna Instruments, Eibar, Spain) equipped with a penetration glass probe.

#### 2.3.3. Cooking Loss and Texture Profile Analysis

For analysis of cooking loss and instrumental texture, the burgers were cooked using vacuum package bags and introduced in a water bath with automatic temperature control (JP Selecta, Precisdg, Barcelona, Spain) until they reached an internal temperature of 70 °C, monitoring the heat by a thermocouples type K (Comark, PK23M, St Neots, UK) connected to a data logger (Comark Dilligence EVG, N3014). Cooking loss was measured by difference in weight between cooked and raw samples.

Texture profile analysis (TPA) (hardness, springiness, cohesiveness, gumminess and chewiness) was measured by compressing to 60% (cylindric probe with flat surface area of 19.85 cm^2^) and the force-time curves were recorded at 3.33 mm/s crosshead speed. The texture parameters were obtained using Texture Exponent 32 software (version 1.0.0.68, StableMicro Systems, Vienna Court, UK).

#### 2.3.4. Lipid Oxidation

The thiobarbituric acid reactive substances (TBARS) index (2-thiobarbituric acid; secondary products of the lipid oxidation) was determined [40] to assess the degree of lipid oxidation in the different samples. Thiobarbituric acid reactive substances (TBARS) values were calculated from a standard curve of malonaldehyde (MDA) with 1,1,3,3-tetraethoxipropane (TEP) and expressed as mg MDA/kg sample.

### 2.4. Fatty Acid Analysis of Deer Burger

For fatty acid analysis, total fat was extracted following the method described by Bligh and Dyer [41] with the modifications proposed by Barros et al. [31].

The fatty acids were transesterified according to the procedure previously described by Barros et al. [31]: for the fatty acids analysis, twenty milligrams of extracted fat dissolved in 1 mL of toluene were transesterified with sodium methoxide and H_2_SO_4_-methanol solution. For the extraction of fatty acid methyl esters, 1 mL of hexane was added to the samples and the organic phase was then transferred to an appropriate GC vial.

Separation and quantification of fatty acids methyl esters (FAMEs) were carried out using a gas chromatograph (GC-Agilent 7890B, Agilent Technologies, Santa Clara, CA, USA) equipped with a flame ionization detector (FID) and PAL RTC-120 auto sampler. For the separation of FAMEs, a DB-23 fused silica capillary column (60 m, 0.25 mm i.d., 0.25 μm film thickness; Agilent Technologies) was used. Chromatographic conditions were completely detailed in the a previous manuscript [31]. Individual FAMEs were identified by comparing their retention times with those of authenticated standards (FAME Mix-37 components; docosapentaenoic acid (C22:5n-3; DPA); *trans*-11 vaccenic acid (11t-C18:1; TVA); *cis*-vaccenic acid (C18:1n-7; CVA) (Supelco, Madrid, Spain) and conjugated linoleic acid (9c,11t-C18:2; CLA) (Matreya)) and the results were expressed as g/100 g of total fatty acids identified.

### 2.5. Sensorial Acceptance Evaluation of Deer Burgers

Sensory analysis was conducted by 68 consumers (with ages between 29 and 40 y and from both genders) from Ourense (Spain). The treatments were evaluated in raw and cooked samples, to determine whether the panelist liked or disliked the different batches of fat replacement by vegetable oil emulsions in relation to control. Consumers evaluated the deer burgers by the acceptance test using a 7-point hedonic scale, which ranged from “1—disliked much” to “7—liked much”, for the following attributes: raw burgers (visual aspect and odor) and cooked burgers (odor cooked burger, flavor, juiciness, fibrous, greasy character and overall acceptability). The burgers were cooked in an oven (Rational Combi Master^®^ Plus CMP61, Landsberg am Lech, Germany) equipped with a core temperature probe, until they reached an internal temperature of 70 °C. The samples were cut in 2 cm^3^ portions, which were individually wrapped in foil and marked with a random 3-digit code. The samples were kept warm in a heater at 55 °C until the testing (up to 30 min). To avoid the possible effects of the order of presentation, the samples were presented to panel members in a random order [42] and served to panelist together with water and toast.

### 2.6. Statistical Analysis

Statistical analyses were performed using the SPSS statistical software, version 19 (IBM). Normal distribution and homogeneity of variance were previously tested (Shapiro–Wilk). Data were submitted for analysis of variance (ANOVA) and Tukey test, when ANOVA had a significant effect (*p* < 0.05). For proximate composition, physicochemical analysis and fatty acids data, treatments were considered as fixed effects and replications (the whole experiment was repeated twice) as a random effect, whereas for sensory acceptance, consumers were additionally included in the model as a random effect (each panelist tasted four samples, one from each formulation, in a single session).

## 3. Results and Discussion

The perception that meat products are good sources of nutrients is gradually giving way to a more negative view, perceived as unhealthy by consumers mainly by the presence of unhealthy constituents in its composition (such as high fat, SFA and cholesterol contents). One strategy that may represent an opportunity for the meat industry to improve this perception is the reformulation of meat products using vegetable oils as fat sources [13,34,43,44]. Additionally, the use of deer meat for making burgers is very limited. Thus, the development of this meat product, with an improved nutritional profile, puts a non-commercialized product on the market.

### 3.1. Proximate Composition, Cooking Loss and Texture Parameters of Burgers

The fat replacement by vegetable oils (tiger nut, chia, and linseed) resulted in a significant increase in moisture (*p* < 0.01) and a decrease in protein (*p* < 0.05) and fat contents (*p* < 0.05), whereas the ash content did not show differences among batches (Table 1). Similar results were found by previous studies for moisture in beef burgers reformulated with canola oil [27,45], in beef [8,16,27] and lamb burgers [35] reformulated with chia and/or linseed oil and in beef burgers with tiger nut oil [31] compared to conventional burgers (high animal fat percentage). In contrast, other authors [46] found for tiger nut pork burgers a decrease (*p* < 0.05) in moisture. In the present case, and as occurs in previous studies with partial replacement of animal fat by vegetable oils added as “prosella” emulsion [31], the increase in moisture was due to the amount of water (56 g/100 g), added to prepare the different emulsions.

In contrast, the animal fat replacement resulted in a significant (*p* < 0.05) reduction in protein content. This reduction was also reported by other authors in beef burgers with fat replacement by hydrogelled or prosella emulsions [16,31]. However, other researchers did not find this decrease [8,46,47], while the use of pork skin-canola oil mixture resulted in a significant increase in protein content of beef burgers [27]. In our study, the replacement of animal fat by vegetable oil emulsion caused a decrease in protein content of deer burgers which could be related to the fact that animal back fat contains an important proportion of protein. In fact, the protein content of pork back fat can be around 8% [11], whereas the prosella emulsion contains only water, oil, and gelling agents, resulting in a significant reduction in protein.

In similar way to protein content, lipid percentage also decreased with fat animal replacement. This fact was also reported by several researchers in burgers with fat replacement. Heck et al. [16] found a reduction of fat content (up to 50%) in beef burger with added hydrogelled emulsion with chia and linseed oils as replacement of pork backfat. In addition, Fagundes et al. [27] also reported a fat reduction (~30%) in beef burgers with animal fat replacement by pork skin-canola oil mixture. Moreover, Selani et al. [30] found the same reduction (~50%) in beef burgers with fat replacement by pineapple by-product-canola oil and Alejandre et al. [29] observed the same trend in beef patties reformulated with total replacement of animal fat by gelled emulsion with algae oil (~70% fat reduction). Similarly, in more recent research, Barros et al. [31] also noticed a significant reduction of fat content in beef burgers in which animal fat was replaced by 50% and 100% of tiger nut oil emulsion. In our case, according to the European Regulations [48] all reformulated burgers can be labeled as “low fat” burgers, because they presented a total fat amount below 3 g/100 g of product.

The content of ash did not show significant differences among the deer burgers studied, ranging from 1.90 to 2.02 g/100 g for LIN and TIG batches, respectively. In contrast to our results, other authors find a significant increase in ash in beef burgers with partial and total replacement of animal fat by tiger nut oil emulsion [31], while other researchers, as occurs in the present manuscript, did not find any differences in ash contents between the control formulation and treatments containing sesame oil oleogels as a partial substitute (25% and 50%) of animal fat in beef burgers [28].

Regarding cooking loss, linseed burgers presented the lowest values (23.93%; *p* < 0.05), while the other three batches presented similar values (control (28.31%), tiger nut (27.27%), and chia (27.11%)). Our values are in disagreement with previous studies published by other authors [8,27,28,46] who found different behavior in high-fat burgers, achieving higher cooking loss in the control, with significant (*p* < 0.05) difference when compared to modified burgers. In contrast with our results, the 100% of fat replacement by tiger nut oil emulsion resulted in a significant reduction of cooking loss in beef burgers [31]. Other authors reported, as occurs in the present study, that the replacement of animal fat by chia [16] or canola oil [45] did not modify cooking loss. Cooking loss can be attributed to the high loss of moisture and fat during cooking [46], however both control and reformulated deer burgers were formulated with low fat contents, which can explain that TIG and CHI did not show differences in comparison with control samples. Lower cooking loss values in reformulated burgers with linseed may be occurred because of a higher thermal stability of the microparticles which contributes to moisture retention and suggests that linseed was more effective in retaining moisture after cooking. The higher thermal stability of the linseed microparticles has been demonstrated previously [8].

Texture changes that occur in meat products by healthier fat reformulation are important challenges, especially due to its importance in sensory attributes [8]. However, in the present study the substitution of animal fat by vegetable oils did not affect textural parameters (Table 1). The present results agree with a recent study in which partial (50%) and total (100%) animal fat replacement by tiger nut oil did not modify texture [31]. In this study, authors also use “prosella” emulsion in the reformulation of beef burgers. However, other authors reported an increase in hardness [8,16,27,28], cohesiveness [8,45], chewiness [16,27,45,46] and gumminess [27] in reformulated batches. Replacement of animal fat commonly resulted in a firmer cooked product because lower fat globules of vegetable oils increase protein–protein and protein–lipid interaction, enhancing the resistance to compression and consequently requiring more work to masticate until swallowing [8,45,49]. Probably, in our study this phenomenon did not occur among treatments due to the similar fat content among all batches that causes an analogous protein:lipid ratio. This higher ratio could modify the protein network (aggregated matrix) which results in a harder and more cohesive texture [50]. Hence, texture results can be considered satisfactory from the point of view of lipid reformulation since no texture modifications were observed compared to an animal fat source. Differences between studies may be due to the amount of animal fat replaced, percentage and type of vegetable oil studied, as well as the method of vegetable oil incorporation to the product [45].

### 3.2. pH and Colour Parameters of Burgers During Refrigerated Storage

The pH values (Table 2) only showed significant (*p* < 0.01) differences among batches on day 6 of the experiment, with control and linseed presenting numerically the lowest and highest values (5.72 vs. 5.84), respectively. The pH values significantly (*p* < 0.001) decreased, except for day 6 that had a slight increase, in all batches during the whole display (18 days). Our pH results agree with data reported by previous studies with fat replacement [8,16,27,45,46], which published that lipid reformulation did not cause a great impact on pH values. However, our results (~5.42) were lower than the range of 5.77–6.35 normally found for this meat product according to the aforementioned studies. Nonetheless, other authors reported lower values in control than in reformulated samples [8,31].

Concerning color parameters, fat source did not affect lightness (L*) and yellowness (b*) of deer burgers, since no significant (*p* < 0.05) differences were observed among batches after elaboration. In addition, L* and b* values of control batch did not present significant differences during the whole display (Table 2). On the contrary, all color parameters were significantly affected in deer burgers prepared with vegetable oils during the refrigerated period (18 days). The most affected color parameter was redness (a*), which showed a reduction around 50% (*p* < 0.001) over display. After the manufacturing process (day 0), the highest a* value was observed in LIN group (12.86), while on day 18 the highest value (6.33) was observed for CHI treatment. The decrease in a* values during storage time are explained by the oxidation of the iron atom within the heme group and the formation of MetMb [51]. The reduction of a* values during storage was reported by several authors in pork [52,53], sheep [54,55,56,57,58], lamb [35] and beef burgers [57].

No significant difference (*p* > 0.05) in L* values among batches was observed. This fact could be due to the already discussed texture parameters (i.e., protein:fat ratio), since L* values are influenced by physical phenomena of light scattering in a heterogeneous food products [28]. Our results are in agreement with data reported by other authors who did not find differences in L* [45] and b* [8,27,28,45] values in meat products since the vegetable oil were utilized as partial or total replacement. On the contrary, aforementioned authors also found no significant differences in a* values. In addition, beef burgers with fat substitution showed a more yellowness (higher b* values) than the control [16,31]. However, other authors found significant differences (*p* < 0.05) for all color parameters between batches (conventional pork burger vs. tiger nut burger) [46]. Differences in instrumental color values between the control and the treatments (ΔE) were >2, which means that the differences identified by instrument are unlikely to be noticed by consumers [16].

### 3.3. Lipid Oxidation (TBARS) of Burgers during Refrigerated Storage

**Figure 1:**
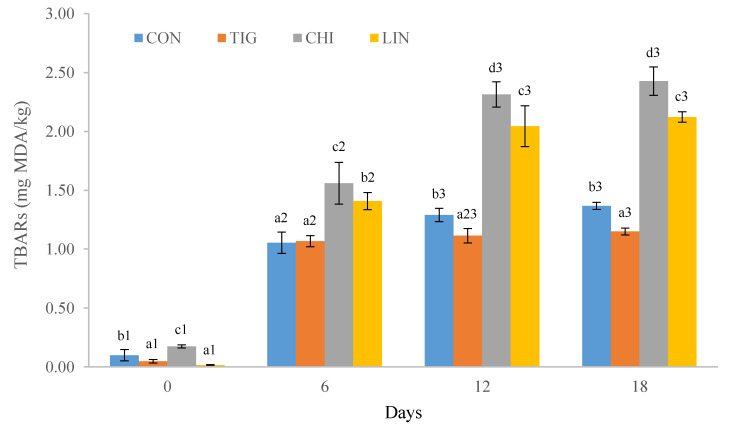
Thiobarbituric acid reactive substances (TBARS) values of the different formulation burgers. ^a–d^ Mean values (different batch in the same day) with different letters indicate significant difference (*p* < 0.05); ^1–3^ Mean values (same batch in different days) with different numerals indicate significant difference (*p* < 0.05). Treatments: CON: burgers prepared 100% pork fat; TIG: burgers prepared with 100% of pork fat replaced by tiger nut oil; CHI: burgers prepared with 100% of pork fat replaced by chia oil; LIN: burgers prepared with 100% of pork fat replaced by linseed oil.

The effect of fat substitution on lipid oxidation (evaluated by TBARS index) of deer burgers is shown in Figure 1. Results displayed a significant effect (*p* < 0.001) by vegetable oil reformulation. Burgers elaborated with chia presented the highest values, followed by control, tiger nut, and linseed (0.20 > 0.08 > 0.05 > 0.01 mg MDA/kg, respectively). TBARS values in deer burgers prepared with tiger nut and linseed presented no significant differences between them. Similarly, other authors reported in a recent study that both partial and total animal fat replacement by tiger nut oil emulsion resulted in a significantly lower TBARs values than control samples. Then, during the refrigerated period, the TBARs values of LIN and CHI batches were higher than CON and TIG treatments. This outcome is in accordance with data found by other authors who observed higher levels for lipid oxidation in meat products elaborated with chia, followed by linseed, tiger nut and pork backfat [9].

In all batches, the TBARS values increased (*p* < 0.001) during the whole display (18 days), showing the highest TBARS values at the end of storage period (1.37, 1.15, 2.43 and 2.12 mg MDA/kg, for CON, TIG, CHI and LIN batches, respectively). Lipid deterioration could be related with the susceptibility of PUFA-rich fats to oxidation [8,9,58], considering that in the present study burgers with chia oil had 35.42% more PUFA than linseed oil, and 306.25% more PUFA than tiger nut oil. All batches were kept below the limit for the oxidation acceptability of 2.5 mg MDA/kg [59]. However, other authors noted that rancid flavor deterioration could be detected at TBARS values higher than 0.6 mg MDA/kg [60]. Taking into account this threshold limit, from day 6 of the experiment all batches exceeded this deterioration level of rancid flavor. With the aim of solving high lipid oxidation, previous studies proposed the addition of natural antioxidants in the products to prevent the oxidative degradation, increasing oxidative stability of rich n-3 PUFA oils, simultaneously reducing volatile compounds derived from oxidation reactions [9,35,47,61,62]. Our TBARS values are lower than those described by de Carvalho et al. [35] who reported that TBARS values in lamb burgers reformulated with chia emulsion ranged between 7 and 10 mg MDA/Kg after 18 days of refrigerated storage. These differences could be related with the fact that these authors formulated burgers with higher fat content (6%–7%) [35] than our burgers, which results in higher lipid oxidation.

### 3.4. Fatty Acid Profile of Burgers

The reformulation significantly improved the fatty acid profile of deer burgers (Table 3). In control samples the main fatty acids were SFA (40.63 g/100 g FA), followed by MUFA (40.58 g/100 g FA), and PUFA (18.79 g/100 g FA). A different fatty acid profile was observed in burgers formulated with tiger nut, in which MUFA was the most abundant fatty acids (52.18 g/100 g FA), followed by SFA (31.48 g/100 g FA) and PUFA (16.34 g/100 g FA). As expected, in samples from the other two batches, the most abundant fatty acids were PUFA in burgers prepared with chia (PUFA > SFA > MUFA; 65.54 > 19.23 > 15.23 g/100 g FA) and linseed (PUFA > SFA > MUFA; 48.17 > 27.04 > 24.79 g/100 g FA) oils. These results are in agreement with data reported by previous studies with beef burgers [8,16].

Reformulation with chia and linseed oils resulted in a fatty acid profile modified compared to control treatment, decreasing the total amount of SFA in deer burgers by 52.50% (CHI group) and 32.50% (LIN treatment), as well as increasing PUFA by 261.11% (CHI batch) and 166.67% (LIN group). This fact is due to the particular fatty acid composition of these oils. Chia oil had very high α-linolenic acid (C18:3n-3: 63%) and PUFA content (82%). Similarly, the content of C18:3n-3 in linseed oil is about 55% and PUFA reached 70% of total fatty acids [9]. In contrast, pork back fat contained high MUFA (47%) and SFA (~35%) and very low PUFA (18%) and n-3 content (<1.5%) [9,32,63]. Moreover, it is important to note that these increases in PUFA content in deer burgers prepared with chia and linseed was due to the higher (*p* < 0.001) n-3 PUFA contents of these samples in comparison with those observed in the CON treatment (43.77 g/100 g FA vs. 31.68 g/100 g FA vs. 3.04 g/100 g FA, for CHI, LIN and CON treatments, respectively). In addition, higher n-6 PUFA contents were also observed in deer burgers prepared with chia than CON samples. Additionally, another important aspect is that the content of essential fatty acids (C18:2n-6 and C18:3n-3) was ~60 g/100 g FA in CHI group and 43 g/100 g FA in LIN treatment. The deer burgers prepared with chia and linseed oil emulsions showed a reduction of over 30% in the SFA substituted by unsaturated fat content (when compared to the control). In this regard, these burgers can be claimed as “reduced saturated fat” according to the European Regulation [48]. Additionally, the burgers from these two batches can be also claimed as “high content of omega-3” because both types of deer burgers presented higher amounts than the minimum value (0.6 g C18:3n-3/100 g of product) reported in the Regulation [48] (1.06 and 0.79 g C18:3n-3/100 g of product for CHI and LIN treatments, respectively; data not shown).

In contrast, the replacement of animal fat by tiger nut oil decreased PUFA content (18.79 g/100 g FA vs. 16.34 g/100 g FA, for CON and TIG batches, respectively). However, a significant increase in MUFA (30%) and decrease in SFA (22.18%) compared to control was observed. This fact is associated with the higher amount of oleic acid (C18:1n-9) present in TIG burger (46.30 g/100 g FA; Table 3). Thus, fatty acid composition of tiger nut burgers reflects the tiger nut oil fatty acids composition, because this oil had high contents of C18:1n-9 (67 g/100 g FA) [9,17,31].

In quantitative terms, C18:1n-9 > palmitic acid (C16:0) > stearic acid (C18:0) and >linoleic acid (C18:2n-6) were the major fatty acids found in CON and TIG treatments. This profile is in agreement with those reported by other authors in beef burgers manufactured with pork back fat [8]. Moreover, in a previous study, authors also found the same fatty acids profile in control and reformulated beef burgers with tiger nut oil emulsion [31]. On the other hand, C18:3n-3, C18:2n-6, C18:1n-9, C16:0 and C18:0 were the most abundant in CHI and LIN burgers. Thus, as a general conclusion, due to the low fat content of deer meat [25], burgers reflected the fatty acid composition of the fat or oil used in their manufacture. The same conclusions were reported by other authors in reformulated burgers [8,16,27,31,35,45] and also in other meat products as pâté [9,14,63], dry-cured sausages [12,64] and cooked or Frankfurter type sausages [10,13].

Regarding the nutritional value of deer burgers (Table 3), the low n-6/n-3 ratio of CHI and LIN treatments (0.49 and 0.51, respectively) when compared to the CON and TIG batches (5.43 and 4.79, respectively) give an idea of the healthiness of the products due to the high content of n-3 PUFAs [16]. This high C18:3n-3 level in chia and linseed led to an increase (*p* < 0.001) in PUFA levels and a higher PUFA/SFA ratio. Recent studies concluded that the reduction in n-6 and n-6/n-3 ratio is one of the main challenges for the development of healthier meat products, since meat products should have a maximum ratio of 4:1 [16,65,66]. According to the European Food Safety Authority—EFSA [67], there are not sufficient data to define a precise fat intake to SFA, MUFA, PUFA or n-6/n3 ratio. However, several international authorities such as EFSA, FAO and USDA recommended that SFAs intake should be as low as possible [67] or less than 10% of calories (2000 or 2500 calorie diet) by replacing them with MUFAs and PUFAs [68,69]. On the other hand, according to FAO nutritional recommendations [69], the n-6/n-3 ratio should be less than 4.0. Nutritional values obtained in deer burgers reformulated with chia and linseed oil emulsion satisfied advice proposed by the international authorities (EFSA, FAO and USDA) since significant values (*p* < 0.001) of SFA substitution by unsaturated fats (already discussed) or lower levels than those ratios recommended (<4.0) were achieved. On the contrary, both CON and TIG deer burgers exceed aforementioned recommendations by 1.43 and 0.79, respectively.

### 3.5. Sensory Analysis of Burgers

The sensory properties of deer burgers (Table 4) were influenced by the reformulation with vegetable oils. Burgers prepared with tiger nut oil achieved the highest acceptance scores, whereas CHI treatment obtained the lowest (5.3 > 5.0 > 4.9 > 4.0 for TIG, CON, LIN and CHI batches, respectively). It is important to note that, although TIG burgers had the highest overall acceptance, there were no significant differences among deer burgers from TIG, CON and LIN batches, while CHI samples showed significant (*p* < 0.01) lower overall acceptability scores. These findings agree with those reported by Barros et al. [31] who observed that the partial (50%) or total replacement of animal fat by tiger nut oil emulsion resulted in higher acceptance, but no significant differences between batches were found. In a similar way, it was reported that the use of an olive and flaxseed oil mixture as a partial substitute for animal fat did not affect the beef burgers sensory parameters [70]. The same conclusion was reported in a study in which animal fat was partially replaced by pork skin-canola oil gel. These authors reported that 50% of animal fat replacement by this mixture containing 10% or 20% of canola oil did not affect the overall acceptability [27]. Another study verified that the 50% replacement of animal fat by sesame oil oleogels was considered acceptable by consumers [28].

The greater the PUFAs content in the burger (chia) the lower evaluation on sensory attributes it received (interactive effects). Our findings are in agreement with data reported by Heck et al. [8] who observed the lowest sensorial values (unpleasant taste) for beef burgers reformulated with chia oil. These authors found the lowest sensory acceptability in unencapsulated chia and linseed oil samples, due to chia oil samples were correlated with negative descriptors, such as bitter and unpleasant taste and dry and rubbery texture [8]. In contrast, the same authors in a more recent study reported that 60% of animal replacement by hydrogelled emulsion with chia and linseed oils presented a higher acceptability compared to the control burgers [16].

An acceptable differentiation among batches was possible with PCA analysis, in which the first two components accumulated a percentage of variance > 70%. Attribute maps of deer burgers showed 87.30% of total variability, since F1 explained 61.77% and F2 25.54%. The attributes more influenced by F1 were odor (raw and cooked), flavor, hardness, and greasy character, whereas visual aspect and juiciness had higher weight in F2 (Table 5).

**Figure 2:**
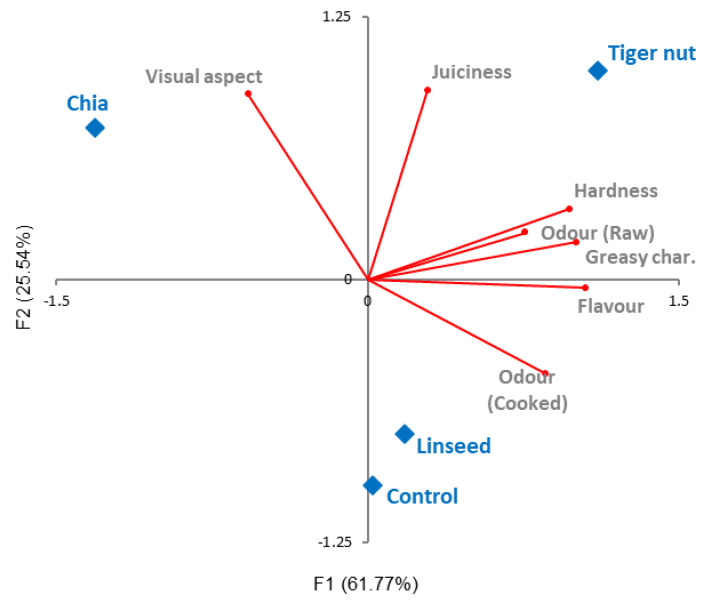
Projection of the sensory attributes and samples batch in the plane defined by the components.

Furthermore, considering F1 and/or the attributes related with this axis (Figure 2), the spatial separation showed that batches were separated in two groups. One of them composed by CON, TIG and LIN batches, and the second one by CHI treatment. Taking into account the results obtained from the sensorial analysis, TIG and LIN deer burgers could be an opportunity for pork backfat replacement as fat source, since its incorporation did not modify the global acceptance.

## 4. Conclusions

Our results demonstrated that the substitution of pork backfat by vegetable oils is a recommendable approach to obtain healthier meat products according to dietary recommendations of the main world agencies, as well growing consumer demand. Reformulation with chia and linseed improved considerably the nutritional value of deer burgers in relation to PUFAs and low n-6/n-3 ratios. Additionally, the samples of these two batches can be claimed as “high content of omega-3” and “low saturated fat” and all reformulated batches could be labelled as “low fat” burgers, which is a great advantage when marketing these deer burgers, as this product is normally perceived by the consumer as a meat product with high fat and saturated fat content. Also, results for texture were positive, considering that reformulation with vegetable oils are a challenge for the meat industry, and the present study did not find significant differences between control and reformulated batches. On the other hand, increase in PUFAs modified the shelf-life of deer burgers, being more susceptible to lipid oxidation, although the TBARS values were below 2.5 mg MDA/kg in all sampling points. From the sensory point of view, the replacement of animal fat by tiger nut or linseed oil emulsion did not affect sensory properties, whereas the use of chia oil reduces the overall acceptability. Moreover, the use of deer meat in combination with tiger nut oil emulsion significantly reduced SFA and increased MUFA, while linseed oil emulsion produced a significant reduction of SFA and n-6/n-3 ratio and increased total and n-3 PUFA in comparison with control samples. Thus, with all results in mind, we can conclude that the use of tiger nut or linseed oil emulsions provides a healthy meat product for the market without reduced physiochemical or sensory properties.

## Figures and Tables

**Table 1:** Proximate composition, cooking loss and texture parameters of deer burgers.

Parameters	Treatments				SEM	Sig.
	CON	TIG	CHI	LIN		
***Proximate composition (%)***						
Moisture	74.50 ^a^	75.27 ^b^	75.66 ^b^	75.94 ^b^	0.16	**
Fat	3.64 ^b^	2.10 ^a^	2.57 ^a^	2.66 ^a^	0.10	**
Protein	19.04 ^b^	18.51 ^a^	18.46 ^a^	18.51 ^a^	0.09	*
Ash	1.99	2.02	1.97	1.90	0.03	n.s.
***Cooking loss (%)***	28.31 ^b^	27.27 ^b^	27.11 ^b^	23.93 ^a^	0.59	*
***TPA test***						
Hardness (N)	88.30	90.34	87.65	80.13	1.80	n.s.
Springiness (mm)	0.72	0.72	0.73	0.76	0.01	n.s.
Cohesiveness	0.60	0.59	0.60	0.61	0.00	n.s.
Gumminess (N)	53.68	53.44	52.75	48.12	1.17	n.s.
Chewiness (N·mm)	39.10	39.78	39.08	35.88	0.95	n.s.

^a,b^ Mean values with different letters indicate significant difference (*p* < 0.05); SEM: standard error of the mean; Sig.: Significance: n.s.: Not significant; ** *p* < 0.01; * *p* < 0.05. Treatments: CON: burgers prepared 100% pork fat; TIG: burgers prepared with 100% of pork fat replaced by tiger nut oil; CHI: burgers prepared with 100% of pork fat replaced by chia oil; LIN: burgers prepared with 100% of pork fat replaced by linseed oil.

**Table 2:** pH and color parameters of deer burgers during refrigerated storage.

Parameters	Days	Treatments				SEM	Sig.
		CON	TIG	CHI	LIN		
pH	0	5.43 ^2^	5.43 ^2^	5.36 ^2^	5.48 ^2^	0.020	n.s.
	6	5.72 ^a3^	5.79 ^bc3^	5.73 ^ab3^	5.84 ^c3^	0.014	**
	12	5.27 ^1^	5.31 ^1^	5.27 ^1^	5.28 ^1^	0.012	n.s.
	18	5.25 ^1^	5.28 ^1^	5.24 ^1^	5.22 ^1^	0.012	n.s.
	SEM	0.045	0.050	0.047	0.056		
	Sig.	***	***	***	***		
L*	0	36.64	33.54 ^1^	34.74 ^1^	33.86 ^1^	0.479	n.s.
	6	35.36	34.58 ^1^	34.83 ^1^	34.28 ^1^	0.305	n.s.
	12	38.43	38.60 ^2^	39.26 ^2^	39.35 ^2^	0.290	n.s.
	18	38.09	39.07 ^2^	38.83 ^2^	38.01 ^2^	0.338	n.s.
	SEM	0.501	0.619	0.575	0.610		
	Sig.	n.s.	***	***	***		
a*	0	10.84 ^a3^	11.97 ^b3^	12.50 ^b3^	12.86 ^b3^	0.225	**
	6	6.82 ^a2^	7.98 ^b2^	8.30 ^b2^	7.66 ^b2^	0.177	**
	12	5.35 ^a1^	5.84 ^ab1^	6.35 ^b1^	6.25 ^b1^	0.147	*
	18	5.03 ^a1^	5.72 ^ab1^	6.33 ^c1^	6.05 ^c1^	0.167	*
	SEM	0.552	0.590	0.594	0.641		
	Sig.	***	***	***	***		
b*	0	12.34	11.32 ^1^	11.77 ^1^	12.41 ^1^	0.204	n.s.
	6	13.08	12.35 ^2^	13.19 ^12^	12.70 ^1^	0.203	n.s.
	12	13.55	13.56 ^3^	13.83 ^2^	14.77 ^2^	0.271	n.s.
	18	13.69	13.88 ^3^	13.56 ^2^	13.90 ^12^	0.213	n.s.
	SEM	0.227	0.266	0.320	0.310		
	Sig.	n.s.	***	***	*		
ΔE*	0–6	6.28 ^c^	5.05 ^ab1^	4.67 ^a1^	5.75 ^b1^	0.213	*
	0–12	7.09 ^a^	8.70 ^b2^	8.86 ^b2^	8.89 ^b2^	0.230	**
	0–18	6.26 ^a^	8.52 ^c2^	7.48 ^b2^	8.19 ^bc2^	0.271	**
	SEM	0.268	0.467	0.506	0.403		
	Sig.	n.s.	***	***	***		

^a–d^ Mean values in the same row (different batch in the same day) with different letters indicate significant difference (*p* < 0.05); ^1–3^ Mean values in the same column (same batch in different days) with different numerals indicate significant difference (*p* < 0.05); SEM: standard error of the mean; Sig.: Significance; n.s.: Not significant; * *p* < 0.05; ** *p* < 0.01; *** *p* < 0.001. Treatments: CON: burgers prepared 100% pork fat; TIG: burgers prepared with 100% of pork fat replaced by tiger nut oil; CHI: burgers prepared with 100% of pork fat replaced by chia oil; LIN: burgers prepared with 100% of pork fat replaced by linseed oil; L*: Lightness; a*: redness; b*: yellowness; ΔE*: total color difference.

**Table 3:** Effect of fat replacement on fatty acid profile of deer burger (g/100 g of total fatty acids).

	Treatment				SEM	Sig.
	CON	TIG	CHI	LIN		
C14:0	1.89 ^a^	1.25 ^b^	0.57 ^c^	1.80 ^a^	0.134	***
C14:1n-5	0.37 ^b^	0.31 ^b^	0.14 ^c^	0.60 ^a^	0.045	***
C15:0	0.29 ^a^	0.34 ^a^	0.16 ^b^	0.31 ^a^	0.020	***
C15:1n-5	1.09 ^b^	1.09 ^b^	1.98 ^a^	1.22 ^b^	0.104	***
C16:0	21.29 ^a^	15.40 ^b^	8.09 ^d^	12.91 ^c^	1.108	***
C16:1n-7	2.87 ^a^	1.83 ^b^	0.77 ^c^	2.54 ^a^	0.198	***
C17:0	0.50 ^a^	0.41 ^b^	0.24 ^c^	0.36 ^b^	0.024	***
C17:1n-7	0.27 ^a^	0.13 ^b^	0.08 ^c^	0.12 ^b^	0.016	***
C18:0	16.28 ^a^	12.90 ^b^	9.51 ^d^	11.23 ^c^	0.597	***
9t-C18:1	0.29 ^a^	0.21 ^b^	0.12 ^c^	0.19 ^b^	0.014	***
11t-C18:1	0.75 ^a^	0.69 ^a^	0.30 ^b^	0.71 ^a^	0.052	***
C18:1n-9	31.68 ^b^	46.30 ^a^	10.70 ^d^	17.63 ^c^	3.161	***
C18:1n-7	2.60 ^a^	1.39 ^c^	1.01 ^d^	1.61 ^b^	0.138	***
C18:2n-6	12.35 ^c^	10.55 ^d^	18.23 ^a^	13.38 ^b^	0.668	***
C18:3n-3	1.38 ^c^	1.08 ^c^	41.31 ^a^	29.85 ^b^	4.059	***
9c,11t-C18:2	0.16 ^a^	0.13 ^a^	0.08 ^b^	0.14 ^a^	0.009	***
C20:0	0.27 ^c^	0.68 ^a^	0.37 ^b^	0.19 ^d^	0.043	***
C20:1n-9	0.64 ^a^	0.19 ^b^	0.14 ^b^	0.15 ^b^	0.051	***
C20:2n-6	0.41 ^a^	0.06 ^c^	0.12 ^b^	0.07 ^c^	0.033	***
C20:3n-6	0.27 ^b^	0.30 ^ab^	0.36 ^a^	0.25 ^b^	0.016	*
C20:4n-6	2.42	2.38	2.71	2.52	0.123	n.s.
C20:5n-3	0.43 ^b^	0.46 ^b^	0.77 ^a^	0.47 ^b^	0.040	***
C24:0	n.d. ^d^	0.27 ^a^	0.12 ^b^	0.07 ^c^	0.023	***
C22:5n-3	0.97	1.03	1.38	1.09	0.063	n.s.
C22:6n-3	0.15	0.17	0.20	0.19	0.009	n.s.
SFA	40.63 ^a^	31.48 ^b^	19.23 ^d^	27.04 ^c^	1.801	***
MUFA	40.58 ^b^	52.18 ^a^	15.23 ^d^	24.79 ^c^	3.280	***
PUFA	18.79 ^c^	16.34 ^c^	65.54 ^a^	48.17 ^b^	4.748	***
n-3	3.04 ^c^	2.80 ^c^	43.77 ^a^	31.68 ^b^	4.122	***
n-6	15.46 ^b^	13.28 ^c^	21.61 ^a^	16.21 ^b^	0.747	***
n-6/n-3	5.43 ^a^	4.79 ^a^	0.49 ^b^	0.51 ^b^	0.549	***

^a–d^ Mean values in the same row (corresponding to the same parameter) with different letter differ significantly (*p* < 0.05; Duncan test); n.d.: not detected; SEM: Standard error of the mean. Sig.: Significance; n.s.: Not significant; * *p* < 0.05; *** *p* < 0.001. Treatments: CON: burgers prepared 100% pork fat; TIG: burgers prepared with 100% of pork fat replaced by tiger nut oil; CHI: burgers prepared with 100% of pork fat replaced by chia oil; LIN: burgers prepared with 100% of pork fat replaced by linseed oil.

**Table 4:** Acceptance scores of burger reformulation with vegetable oils.

	Acceptance Scores							
	VA	OR	OC	FL	JU	FI	GC	OA
**CON**	5.3	5.1	5.4	5.0	4.9	5.0	4.9	5.0 ^b^
**TIG**	5.5	5.1	5.6	5.4	5.2	5.1	5.1	5.3 ^b^
**CHI**	5.8	4.7	4.6	4.3	5.2	4.8	4.8	4.0 ^a^
**LIN**	5.3	4.6	5.2	5.1	5.1	4.9	5.0	4.9 ^b^
**F**								4.215
**Sig.**								**

^a,b^ Mean values with different letters indicate significant difference (*p* < 0.05). Sig.: Significance; ** *p* < 0.01. Treatments: CON: burgers prepared 100% pork fat; TIG: burgers prepared with 100% of pork fat replaced by tiger nut oil; CHI: burgers prepared with 100% of pork fat replaced by chia oil; LIN: burgers prepared with 100% of pork fat replaced by linseed oil. VA: visual aspect (raw burger); OR: odor (raw burger); OC: odor cooked burger; FL: flavor; JU: juiciness; FI: fibrous; GC: greasy character; OA: overall acceptability.

**Table 5:** Sensory properties correlation with F1 and F2 of PCA.

	F1	F2
**VA**	0.298	***0.699***
**OR**	***0.523***	0.045
**OC**	***0.664***	0.181
**FL**	***0.998***	0.001
**JU**	0.075	***0.732***
**FI**	***0.851***	0.101
**GC**	***0.913***	0.028

Treatments: CON: burgers prepared 100% pork fat; TIG: burgers prepared with 100% of pork fat replaced by tiger nut oil; CHI: burgers prepared with 100% of pork fat replaced by chia oil; LIN: burgers prepared with 100% of pork fat replaced by linseed oil. VA: visual aspect; OR: odor (raw burger); OC: odor cooked burger; FL: flavor; JU: juiciness; FI: fibrous; GC: greasy character. The attributes highlighted in italics and bold had more influence on F1 or F2 axis.
